# Bronchiectasis Exacerbation Increases the Risk of Adverse Renal Outcomes—Results From a Large Territory‐Wide Cohort Study

**DOI:** 10.1111/crj.70029

**Published:** 2025-01-11

**Authors:** Wang Chun Kwok, Chung Ki Tsui, Leung Sze Him Isaac, Chun Ka Emmanuel Wong, Terence Chi Chun Tam, James Chung Man Ho, Desmond Yat Hin Yap

**Affiliations:** ^1^ Division of Respiratory Medicine and Critical Care Medicine, Department of Medicine, Queen Mary Hospital The University of Hong Kong Pok Fu Lam Hong Kong SAR China; ^2^ Department of Statistics The Chinese University of Hong Kong Sha Tin Hong Kong SAR China; ^3^ Division of Nephrology, Department of Medicine, Queen Mary Hospital The University of Hong Kong Pok Fu Lam Hong Kong SAR China

**Keywords:** acute kidney injury, bronchiectasis, chronic kidney disease, exacerbation, renal progression

## Abstract

**Introduction:**

Bronchiectasis exacerbation (BE) is associated with unfavorable sequelae in other organs such as the cardiovascular system; data regarding its impact on adverse term renal outcomes, however, is lacking.

**Methods:**

A territory‐wide retrospective cohort study was conducted in Hong Kong between 1/1/1993 and 31/12/2017. All patients with bronchiectasis followed in the public healthcare system in 2017 were classified as “Exacerbators” or “Non‐Exacerbators,” and their adverse renal outcomes (renal progression [decrease in eGFR by 30 mL/min lasted for more than 12 months during follow up], acute kidney injury [AKI], and annual rate of eGFR decline) in the ensuing 7 years were compared. Results were also analyzed in the 1:1 propensity score matched (PSM) cohort.

**Results:**

A total of 7929 patients (1074 “Exacerbators” group and 6855 “Non‐exacerbators”) were followed for 6.2 ± 1.6 years. A total of 1570 patients (19.8%) had renal progression, and 935 (11.8%) patients developed AKI. “Exacerbators” showed significantly increased risk of renal progression (adjusted odds ratio [aOR] 1. 27 [95% CI 1.08–1.50, *p* = 0.003]), more rapid eGFR decline (−3.67 [−1.74 to −6.54] vs. −3.03 [−1.56 to −5.12] mL/min/1.73 m^2^/year, *p* = 0.004) and AKI (aOR 1.99; 95% CI 1.44–2.73, *p* < 0.001) than the “Non‐exacerbators.” Annual number of BE was associated with renal progression (aOR 1.45; 95% CI 1.22–1.72, *p* < 0.001) and AKI (aOR 2.00; 95% CI 1.38–2.91, *p* < 0.001). Results were consistent in the analysis with the PSM cohort.

**Conclusions:**

Renal progression and AKI are common among patients with bronchiectasis, and BE is an independent risk factor for adverse renal outcomes.

## Graphical abstract

In this territory‐wide retrospective cohort study, bronchiectasis exacerbation is associated with increased risks of renal progression and acute kidney injury. The findings suggested the potential need for regular monitoring of renal function among bronchiectasis who are exacerbation prone with timely reno‐protective strategies.

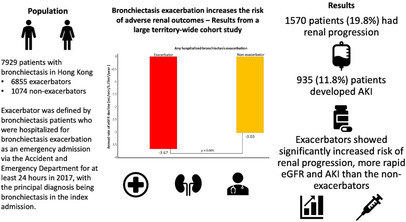

## 1 Introduction

Bronchiectasis is a common suppurative chronic respiratory disease [1, 2]. Bronchiectasis exacerbation (BE) is a common sequalae of bronchiectasis, and the European Multicentre Bronchiectasis Audit and Research Collaboration (EMBARC) Bronchiectasis Registry reported that 50% of patients with bronchiectasis had at least two exacerbations annually [3]. BE is well recognized to confer negative impact on patient's morbidity, quality of life, mortality, and healthcare costs [4, 5, 6, 7, 8, 9, 10, 11, 12].

Adverse systemic outcomes, in particular, cardiovascular events, have been reported to be associated with bronchiectasis [13, 14, 15, 16]. At the same time, chronic respiratory diseases, in particular, in the presence of medical co‐morbidities, can confer detrimental effects on the kidneys [17]. There is limited evidence on the association between bronchiectasis and adverse kidney outcomes. A study reported that 5% of patients hospitalized for BE developed acute kidney injury (AKI), while the development of AKI was associated with up to 33% in‐hospital mortality [18]. Furthermore, data are lacking regarding the impact of BE on adverse renal outcomes. In chronic obstructive pulmonary disease (COPD), exacerbation, in particular, hospitalized COPD exacerbation, was shown to be associated with increased risk of renal progression/death and AKI [19]. In bronchiectasis, there was one only retrospective study that reported outcomes of patients with secondary amyloidosis related to bronchiectasis and end‐stage kidney disease [20]. In view of the above knowledge gaps, we conducted the current study to investigate the relationship between BE and adverse renal outcomes.

## 2 Methods

A territory‐wide retrospective cohort study was conducted in Hong Kong. We retrieved the data for all adult patients with the diagnostic code of bronchiectasis by International Classification of Diseases, Ninth Revision (ICD‐9) code of 494 between January 1, 1993, and December 31, 2017, from Clinical Data Analysis and Reporting System (CDARS) of the Hospital Authority of Hong Kong (HKHA). The patients who were still alive at 1/1/2017 managed in HKHA were included in this study. Patients who had co‐existing asthma, COPD, interstitial lung disease (ILD), and lack of renal function test at baseline or at follow up will be excluded. Baseline characteristics, demographics, clinical data, and outcomes were retrieved from CDARS. HKHA is the major public health care service provider in Hong Kong that operates 43 hospitals and 123 outpatient clinics, serving > 90% of the population in Hong Kong. CDARS is an electronic healthcare database managed by the HKHA, which consisted of all essential clinical data (patient demographics, hospitalization record, outpatient clinics and emergency departments attendance, diagnoses, laboratory test results, procedures performed, medication record, death date, and cause of death) prospectively recorded. To protect patients' confidentiality, a unique, anonymous reference key is assigned to each individual patient that is linked to all their clinical information in CDARS. The positive predictive value of the diagnostic code bronchiectasis in CDARS was reported to be 92.7% [21].

The “Exacerbator” group was defined by bronchiectasis patients (diagnosis code ICD‐9: 494) who were hospitalized for BE as an emergency hospital admission for at least 24 h in year 2017, with the principal diagnosis being “bronchiectasis” in the index admission, and had systemic (oral or intravenous) antibiotics prescribed, among all patients with bronchiectasis on follow‐up in the year 2017. The comparator group was bronchiectasis patients without hospitalized BE. The primary outcome of the study was the development of renal progression upon follow up. Renal progression was defined as persistent decrease in estimated glomerular filtration rate (eGFR) > 30 mL/min/1.73 m^2^ during the follow‐up period, which lasted for > 12 months [22]. The secondary outcomes included the development of AKI and rate of eGFR decline. Patients who lost to follow up were identified by their last available attendance record from CDARS, and they were censored in the study. The censoring date was set as the date with the last data entry on CDARS. The study was approved by the Institutional Review Board of the University of Hong Kong and HKHA West Cluster (UW 22‐763).

### 2.1 Patient and Public Involvement

As the current study is a retrospective territory‐wide study conducted using electronic medical record, it did not involve active patient recruitment.

### 2.2 Statistical Analysis

The statistical analyses were performed using R V.4.2.2 (R Foundation for Statistical Computing) and 28th version of IBM SPSS statistical package. Continuous variables were expressed as mean ± standard deviation (SD) or median and inter‐quartile range (IQR). Mann–Whitney *U*‐test or independent *t*‐test were used to compare the continuous variables of two groups. *χ* ^2^ test or Fisher's exact test were applied for categorical variables.

The association between hospitalized BE and adverse renal outcomes were first assessed by univariate analysis, followed by multi‐variate analysis that adjusted for potential confounders including age, race, sex, underlying hypertension (HT) and diabetes mellitus (DM), history of BE during 2012–2016, *Pseudomonas aeruginosa* ( *P. aeruginosa* ) colonization, baseline eGFR, baseline Charlson comorbidity index (CCI), use of a angiotensin‐converting‐enzyme inhibitors (ACEI), use of angiotensin receptor blocker (ARB), use of nephrotoxic medications, and other factors that were statistically different at baseline. Multivariate linear regression was used to compare the rate of annual eGFR decline in the two groups. Cox regression analysis was used to assess the survival of the patients. Kaplan–Meier estimator was used to assess survival function of the patients in the two groups. To better control for the potential confounders, propensity score adjustment was performed. Patients were matched based on the age, sex, presence of HT and DM, history of BE during 2012–2016, *P. aeruginosa* colonization, baseline eGFR and baseline CCI with 1:1 matching, and caliper of 0.2 times SD of the logit of propensity score. To further reduce the bias from unmeasured confounding, individuals with extreme scores in the upper or lower tail of the propensity score distribution were excluded. Any inadequately matched variables were adjusted in multivariate analysis. Subgroup analysis was performed in patients without known underlying HT and DM, which are the possible risk factors for renal progression and AKI. Statistical significance was determined at the level of *p* = 0.05.

## 3 Results

### 3.1 Patient Characteristics

**FIGURE 1:**
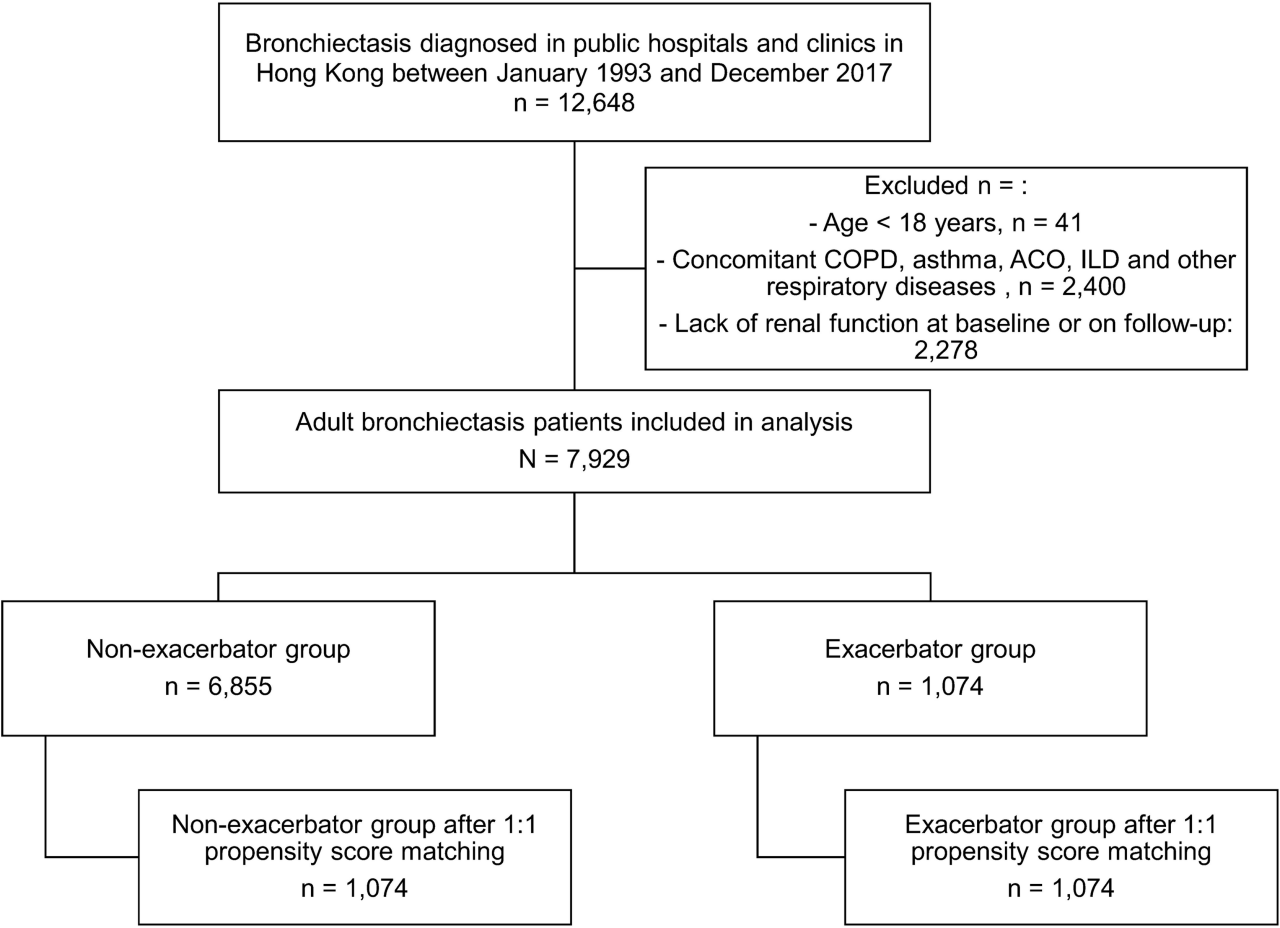
Patient selection flow diagram.

A total of 7929 patients diagnosed with bronchiectasis were included, of whom 1230 had a hospitalized BE in 1074 (exacerbator group) and 6855 did not (non‐exacerbator group). The patient selection flow diagram was illustrated in Figure 1. The mean age of the whole cohort was 69.8 ± 14.0 years, and 44.0% of the patients were men. The mean follow‐up duration was 6.2 ± 1.6 years with data cut‐off at 31/12/2023. The baseline characteristics of the patients were presented in Table 1. Patients in the “Exacerbator” group were older and more likely to have *P. aeruginosa* colonization, past history of BE, HT, DM, hyperlipidemia, congestive heart failure (CHF), and ischemic heart disease (IHD). The “Exacerbator” group also had higher rates of ACEI/ARB prescription and higher bassline neutrophil to lymphocyte ratio (NLR). After propensity score matching, 1074 subjects were included in each group in the analysis (Table 1). The patients were followed up until 31/12/2023.

**TABLE 1:** Baseline demographics of the whole cohort and propensity score matched cohort.

	Whole cohort	Whole cohort		*p*‐value	ASD	Propensity score matched cohort		*p*‐value	ASD
Number of subjects	7929	Exacerbator (*n* = 6855)	Non‐exacerbator (*n* = 1074)			Exacerbator (*n* = 1074)	Non‐exacerbator (*n* = 1074)		
Ethnic groups (*N* (%))				0.93	0.03			0.91	0.04
Chinese	7709 (97.2)	6662 (97.2%)	1047 (97.5%)			1050 (97.8%)	1047 (97.5%)		
South‐East Asian	33 (0.4%)	28 (0.4%)	5 (0.5%)			4 (0.4%)	5 (0.5%)		
South Asian	24 (0.3%)	21 (0.3%)	3 (0.3%)			4 (0.4%)	3 (0.3%)		
Caucasian	34 (0.4%)	31 (0.5%)	3 (0.3%)			4 (0.4%)	3 (0.3%)		
Others	129 (1.6%)	113 (1.6%)	16 (1.5%)			12 (1.1%)	16 (1.5%)		
Age, years (mean ± SD)	69.8 ± 14.0	69.44 ± 14.1	72.26 ± 13.6	< 0.001*	0.20	72.6 ± 13.2	72.3 ± 13.6	0.53	0.03
Male (*N* (%))	3487 (44.0%)	3044 (44.4%)	443 (41.2%)	0.06	0.06	433 (40.3%)	443 (41.2%)	0.69	0.02
Annual number of bronchiectasis exacerbation in year 2012–2016, mean ± SD	0.82 ± 0.38	0.81 ± 0.39	0.86 ± 0.34	< 0.001*	0.14	0.88 ± 0.33	0.86 ± 0.34	0.40	0.04
*Pseudomonas aeruginosa* colonization (*N* (%))	1373 (17.3%)	999 (14.6%)	374 (34.8%)	< 0.001*	0.483	373 (34.7%)	374 (34.8%)	1.00	0.002
Hypertension (*N* (%))	1542 (19.4%)	1241 (18.1%)	301 (28.0%)	< 0.001*	0.24	297 (27.7%)	301 (28.0%)	0.89	0.008
Diabetes mellitus (*N* (%))	663 (8.4%)	542 (7.9%)	121 (11.3%)	< 0.001*	0.11	125 (11.6%)	121 (11.3%)	0.84	0.012
Ischemic heart disease (*N* (%))	561 (7.1%)	456 (6.7%)	105 (9.8%)	< 0.001*	0.114	92 (8.6%)	105 (9.8%)	0.37	0.04
Stroke (*N* (%))	275 (3.5%)	227 (3.3%)	48 (4.5%)	0.07	0.06	45 (4.2%)	48 (4.5%)	0.83	0.01
Heart failure (*N* (%))	401 (5.1%)	311 (4.5%)	90 (8.4%)	< 0.001*	0.157	74 (6.9%)	90 (8.4%)	0.22	0.06
Hyperlipidemia (*N* (%))	457 (5.8%)	381 (5.6%)	76 (7.1%)	0.06	0.06	83 (7.7%)	76 (7.1%)	0.62	0.03
Charlson comorbidity index (mean ± SD)	3.10 ± 1.78	3.04 ± 1.76	3.49 ± 1.83	< 0.001*	0.25	3.51 ± 1.90	3.49 ± 1.83	0.84	0.009
Baseline eGFR, (mL/min/1.73 m ^2^ ) (mean ± SD)	66.60 ± 33.14	66.90 ± 32.36	64.73 ± 37.71	0.05	0.06	63.56 ± 29.87	64.73 ± 37.71	0.43	0.03
Baseline NLR (mean ± SD)	4.76 ± 5.04	4.60 ± 5.05	5.46 ± 4.92	< 0.001*	0.17	4.95 ± 4.91	5.46 ± 4.92	0.03	0.10
Use of ACEI (*N* (%))	1381 (17.4%)	1159 (16.9%)	222 (20.7%)	0.003*	0.096	237 (22.1%)	222 (20.7%)	0.46	0.03
Use of ARB (*N* (%))	825 (10.4%)	693 (10.1%)	132 (12.3%)	0.034*	0.07	143 (13.3%)	132 (12.3%)	0.52	0.03

*Note:* Data are expressed as mean ± S.D.

Abbreviations: ACEI, angiotensin converting enzyme inhibitor; ARB, angiotensin receptor blockers; ASD = absolute standardized difference; eGFR, estimated glomerular filtration rates; mmol = millimoles per liter; NLR = neutrophil to lymphocyte ratio; SD = standard deviation; # = good balance with ASD < 0.1.

* Statistically significant.

### 3.2 Risk Factors for Renal Progression

**FIGURE 2:**
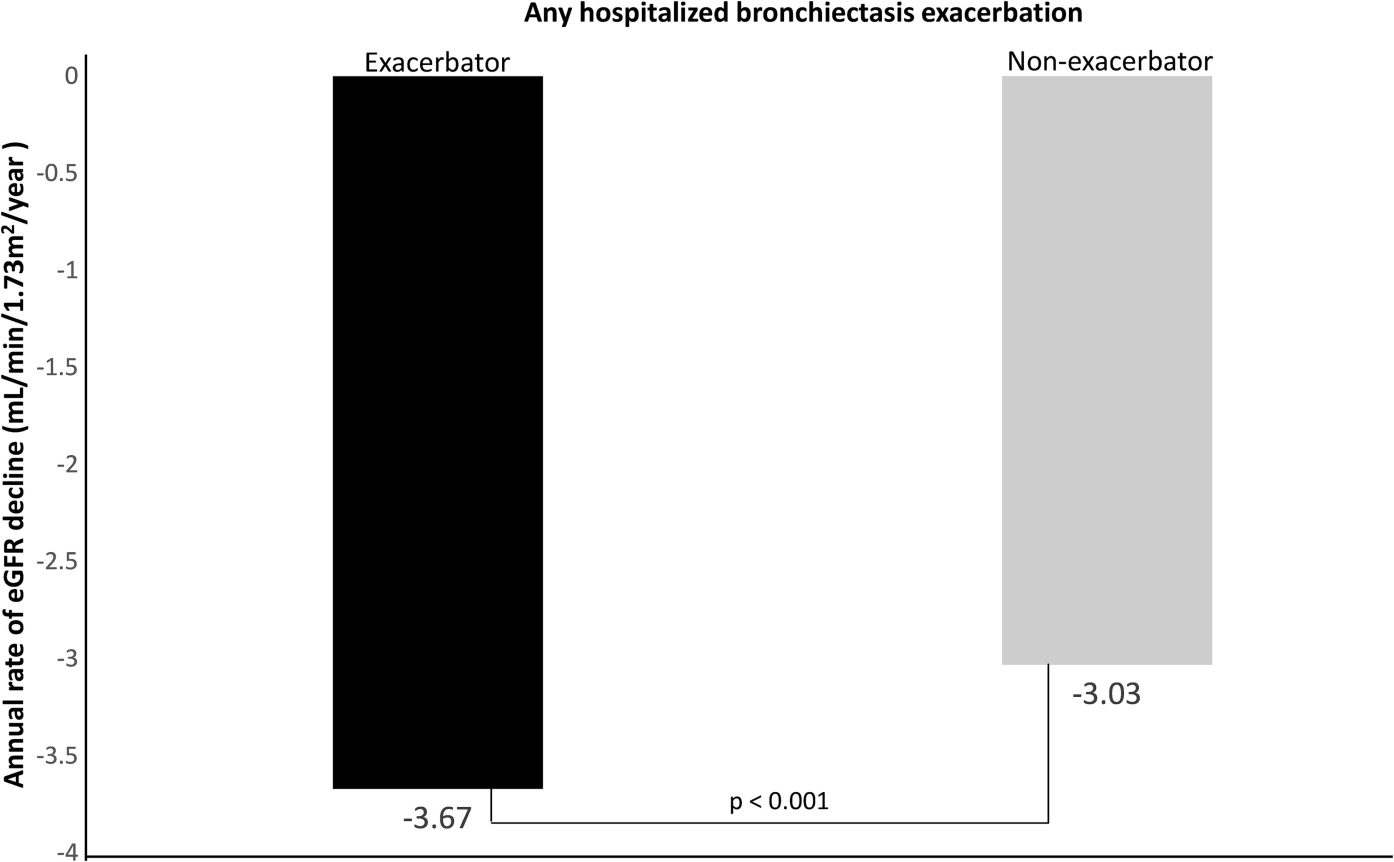
Annual rate of eGFR decline in “Exacerbators” and “Non‐exacerbators” of bronchiectasis.

**FIGURE 3:**
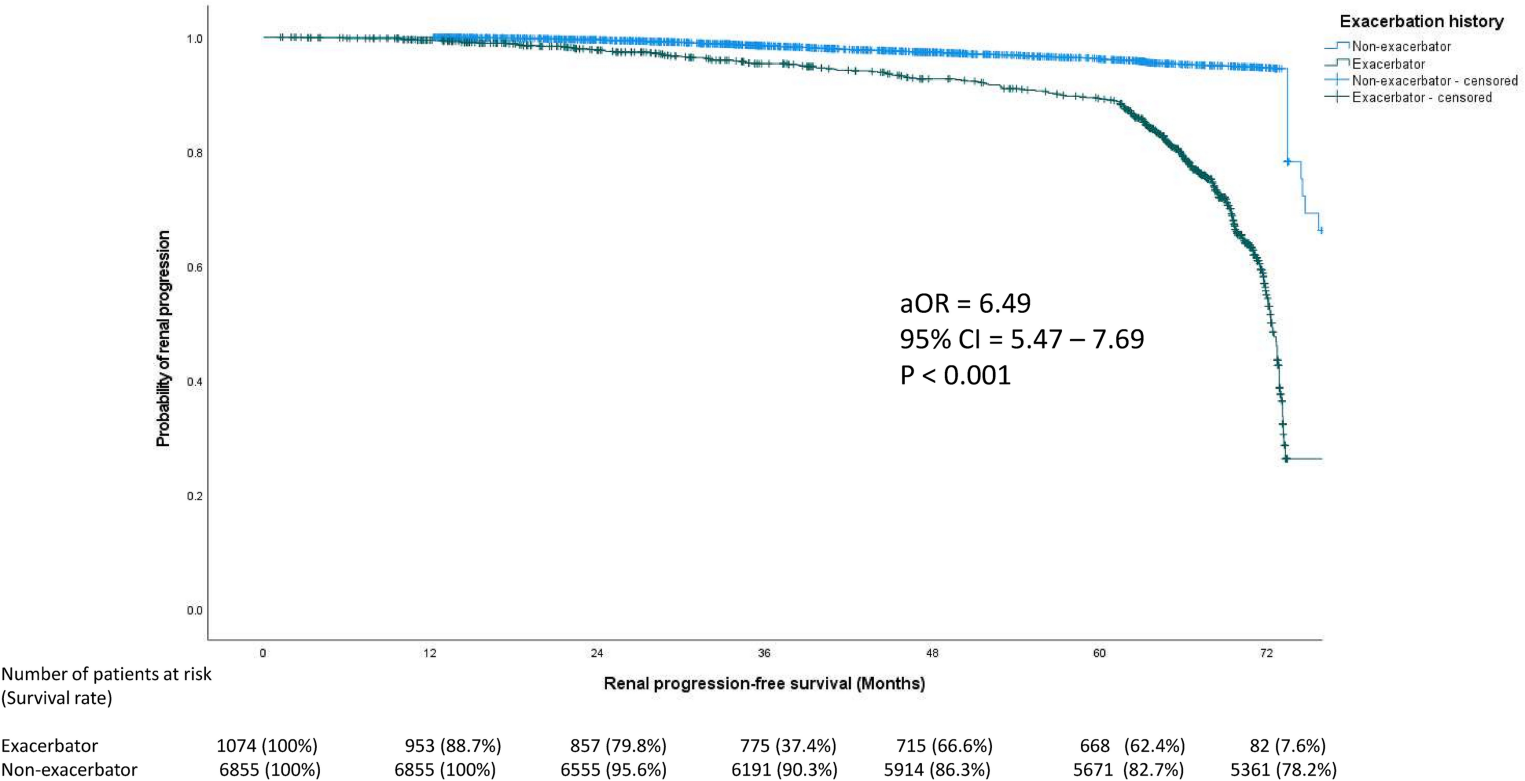
Renal progression‐free survival in “Exacerbators” and “Non‐exacerbators” of bronchiectasis.

A total of 1570 (19.8%) patients developed renal progression during follow‐up, with 273 (25.4%) patients in the “Exacerbator” group and 1297 (18.9%) in the “Non‐exacerbator group” respectively. The “Exacerbator” group showed a more rapid eGFR decline (median annual decline −3.7 (IQR −1.7 to −6.5) mL/min/1.73 m^2^/year versus −3.03 (IQR −1.56 to −5.12) mL/min/1.73 m^2^/year in non‐exacerbator group, with *p* = 0.004) (Figure 2). Univariate analysis showed that “Exacerbator” status, *P. aeruginosa* colonization, IHD, CHF, stroke, HT, DM, baseline CCI, annual number of BE (2012–2016), and low baseline eGFR were all predictors for renal progression (Table 2). In our multivariate analysis, “Exacerbator” status (aOR 1.46; 95% CI 1.26–1.70, *p* = 0.001), annual number of BE (aOR 1.45; 95% CI 1.22–1.72, *p* < 0.001), baseline CCI (aOR 1.30; 95% CI 1.23–1.37, *p* < 0.001), and low baseline eGFR (aOR 1.01; 95% CI 1.00–1.01, *p* < 0.001) remained robust predictors for renal progression. Similar results were found in 1:1 propensity score matched cohort (Table S1). The “Exacerbators” showed significantly worse long‐term renal progression‐free survival compared to “Non‐exacerbators” (aHR 6.49; 95% CI 5.47–7.69, *p* < 0.001) and with shorter median time to renal progression (72.3 months vs. 83.2 months, *p* < 0.001) (Figure 3). In the propensity score matched cohort, the “Exacerbator” group also demonstrated significantly higher risk of renal progression (aOR 1.24; 95% CI 1.01–1.54, *p* = 0.037) and more rapid eGFR decline (−3.7 [IQR −1.74 to −6.54] mL/min/1.73 m^2^/year vs. −3.32 [IQR −1.72 to −6.56] mL/min/1.73 m^2^/year in non‐exacerbator group, *p* = 0.047) than the “Non‐exacerbator” group. Again, the “Exacerbators” group showed significantly inferior long‐term renal progression‐free survival than the “Non‐Exacerbator” group (aHR 4.77; 95% CI 3.69–6.15; *p* < 0.001) (Figure S1).

**TABLE 2:** Risk factors for renal progression in patients with bronchiectasis in the whole cohort.

	Univariate analysis			Multivariate analysis		
	OR	95% CI	*p*‐value	aOR	95% CI	*p*‐value
Exacerbator	1.46	1.16–1.70	< 0.001*	1.46	1.26–1.70	< 0.001*
*Pseudomonas aeruginosa* colonization	1.30	1.16–3.50	< 0.001*	1.06	0.91–1.24	0.43
IHD	1.59	1.31–1.93	< 0.001*	0.77	0.62–0.97	0.024
CHF	1.96	1.57–2.43	< 0.001*	0.91	0.71–1.17	0.46
Stroke	1.73	1.33–2.25	< 0.001*	0.93	0.70–1.25	0.63
HT	1.73	1.52–1.96	< 0.001*	1.14	0.98–1.33	0.085
DM	2.00	1.68–2.38	< 0.001*	0.89	0.73–1.10	0.29
Baseline CCI	1.18	1.15–1.22	< 0.001*	1.30	1.23–1.37	< 0.001*
Annual number of bronchiectasis exacerbation from 2012 to 2016	1.71	1.46–2.02	< 0.001*	1.45	1.22–1.72	< 0.001*
Lower baseline eGFR	1.01	1.00–1.01	< 0.001*	1.01	1.00–1.01	< 0.001*

Abbreviations: CCI = Charlson comorbidity index; CHF = heart failure; CVA = history of stroke; DM = diabetes mellitus; eGFR = estimated glomerular filtration rates; HT = hypertension; IHD = ischemic heart disease.

* Statistically significant.

### 3.3 Risk Factors for AKI

A total of 935 (11.8%) patients developed AKI during follow‐up, with 198 (18.4%) patients in the “Exacerbator” group and 737 (10.8%) in the “Non‐exacerbator” group. Univariate analysis showed that “Exacerbator” status, *P. aeruginosa* colonization, IHD, CHF, stroke, HT, DM, baseline CCI, annual number of BE (2012–2016), and low baseline eGFR were all predictors for AKI (Table 3). In our multivariate analysis, “Exacerbator” status (aOR 1.99; 95% CI 1.44–2.73, *p* < 0.001), annual number of BE (aOR 2.00; 95% CI 1.38–2.91, *p* ≤ 0.001), *P. aeruginosa* colonization (aOR 2.03; 95% CI 1.52–2.71, *p* < 0.001), baseline CCI (aOR 1.22; 95% CI 1.09–1.37, *p* < 0.001), and low baseline eGFR (aOR 1.18; 95% CI 1.16–1.19, *p* < 0.001) remained robust predictors for AKI. Similar results were found in 1:1 propensity score matched cohort (Table S2). In the propensity score matched cohort, the “Exacerbator” group also had significantly higher risk of AKI than the “Non‐exacerbator” group (aOR 2.23; 95% CI 1.45–3.40, *p* < 0.001).

**TABLE 3:** Risk factors for acute kidney injury in patients with bronchiectasis in the whole cohort.

	Univariate analysis			Multivariate analysis		
	OR	95% CI	*p*‐value	aOR	95% CI	*p*‐value
Male	1.39	1.21–1.59	< 0.001*	1.01	0.80–1.27	0.94
Exacerbator	1.58	1.58–2.23	< 0.001*	1.99	1.44–2.73	< 0.001*
*Pseudomonas aeruginosa* colonization	1.97	1.68–2.31	< 0.001*	2.03	1.52–2.71	< 0.001*
IHD	2.52	2.04–3.10	< 0.001*	0.70	0.48–1.03	0.07
CHF	2.37	1.50–3.76	< 0.001*	0.93	1.50–3.76	0.71
Stroke	3.08	2.35–4.05	< 0.001*	1.38	1.38–2.25	0.19
HT	3.13	2.71–3.62	< 0.001*	1.07	0.81–1.39	0.63
DM	3.55	2.95–4.27	< 0.001*	0.56	0.39–0.79	< 0.001*
Baseline CCI	1.54	1.48–1.60	< 0.001*	1.22	1.09–1.37	< 0.001*
Annual number of bronchiectasis exacerbation from 2012 to 2016	2.05	1.65–2.55	< 0.001*	2.00	1.38–2.91	< 0.001*
Lower baseline eGFR	1.16	1.15–1.16	< 0.001*	1.18	1.16–1.19	< 0.001*

Abbreviations: CCI = Charlson comorbidity index; CHF = heart failure; CVA = history of stroke; DM = diabetes mellitus; eGFR = estimated glomerular filtration rates; HT = hypertension; IHD = ischemic heart disease.

* Statistically significant.

### 3.4 Subgroup Analysis

Subgroup analysis was performed among patients without known underlying HT and DM, as these are also possible risk factors for renal progression and AKI. There were 6231 patients in this subgroup among the whole cohort. The “Exacerbator” group had significantly higher risk of AKI than the “Non‐exacerbator” group (aOR 1.34; 95% CI 1.09–1.65, *p* < 0.001), worse long‐term renal progression‐free survival (aHR 5.67; 95% CI 4.58–7.02, *p* < 0.001), and more rapid eGFR decline (−4.70 [IQR −2.47 to −8.77] mL/min/1.73 m^2^/year vs. −6.57 [IQR −3.21 to −12.47] mL/min/1.73 m^2^/year in non‐exacerbator group, *p* < 0.001).

The “Exacerbator” group also had significantly higher risk of AKI than the “Non‐exacerbator” group (aOR 1.69; 95% CI 1.29–2.21, *p* < 0.001).

## 4 Discussion

To our best knowledge, our study was the first to suggest the association between hospitalized BE and adverse renal outcomes. After adjusting for important confounders such as underlying comorbidities and demographic factors, hospitalized BE remains an important independent risk factor for renal progression, annual rate of eGFR decline, and AKI. The results highlight the importance of the negative impact of BE.

BE is an important sequalae of bronchiectasis, which was associated with negative outcomes including higher mortality, morbidity, worse quality of life, and increased healthcare‐related costs [4, 5, 6, 7, 8, 9, 10, 11, 12]. BE was also reported to be associated with adverse cardiovascular outcomes including acute myocardial infarction, CHF, and composite cardiovascular events [23]. This can be related to hypoxia and increased systemic inflammatory mediators at the time of BE. Similar phenomenon was reported for adverse renal outcomes among patients with hospitalized COPD exacerbation [19]. Hospitalized BE, often associated with systemic inflammation and hypoxia, could trigger inflammatory cascade and oxidative stress, which leads to adverse renal outcome. This under‐reported negative outcome of BE warrants the attention from clinicians, to prevent BE as well as monitor renal function among the patients who are at risk of BE.

Furthermore, studies suggested that patients with elevated inflammatory makers at baseline were at risks of BE [24]. Indeed, the “Exacerbators” in this study also had higher baseline NLR although NLR itself could not predict adverse renal outcomes. NLR is a readily available biomarker that reflects the degree of neutrophilic inflammation with prognostic implication in bronchiectasis [25, 26, 27]. The “Exacerbators” group could have heightened systemic inflammation which eventually lead to progressive chronic kidney disease (CKD), with such observation being proposed in many studies [28, 29, 30, 31].

Another point to note is that patients with *P. aeruginosa* colonization were also at risks of renal progression and AKI. In patients with bronchiectasis, *P. aeruginosa* was well reported to have important role in the pathogenesis, inducing inflammation and accelerating disease progression [32]. *P. aeruginosa* colonization has also been suggested to be a risk factor for BE. These could all add up to form a vicious cycle that promotes further lung damage, systemic inflammation, and adverse renal outcomes. To complicate the issue further, the management of *P. aeruginosa* infection often entails the use of nephrotoxic antibiotics (e.g., aminoglycosides), which may precipitate AKI. This includes the long‐term use of inhaled aminoglycoside for those with *P. aeruginosa* colonization and repeated exacerbation [33], as well as the intermittent systemic administration for exacerbation. It is not surprising that lower baseline eGFR was a risk factor for renal progression and AKI, as these patients had less kidney reserve and hence more vulnerable to further nephron damage upon any insults.

While certain medical comorbidity such as DM or HT are common risk factors for AKI or CKD [34, 35, 36], they were not shown to be independent risk factors for adverse kidney outcomes among patients with bronchiectasis in our current study. Instead, our results also showed that higher baseline CCI, a reflection of overall burden of medical comorbidities, was a robust predictor for AKI and renal progression in patients with bronchiectasis.

In the current study, we demonstrated that renal progression and AKI are common in bronchiectasis patients, in particular, those with high exacerbation risks, including the “Exacerbators” group or with history of BE. The finding echoes with prior report of BE and cardiovascular outcomes [23] that BE does not purely affect the lung health but has negative impact in other organ systems. Preventing BE will not only benefit in terms of respiratory function but also on cardiovascular and renal outcomes. In view of these results, clinicians should seriously consider all measures to prevent BE. Eradication of *P. aeruginosa* colonization should seriously be considered [37, 38]. Proper patient phenotyping [39] and personalization of treatment [40] can also potentially improve management of bronchiectasis patients. Last but not least, the standard treatment for bronchiectasis including vaccination [41], maintaining bronchial hygiene [7, 42, 43], and other well‐established pharmacotherapy shall also be considered in appropriate clinical settings [44, 45].

Our study has several limitations to address. First, the study population constituted mostly Chinese patients, which might affect the generalizability of the findings. Nevertheless, it is worthwhile to have a study that predominantly included Chinese patients because the etiology of bronchiectasis in Asian population (in particular Chinese) differs significantly from the Caucasians. Post‐infective bronchiectasis is the commonest cause of bronchiectasis in Asians, while cystic fibrosis is rarely seen in these ethnic groups. As a retrospective study, the number of renal function tests performed, and the timing of renal function tests were not unified. A prospective observational study with unified timing and numbers of renal function assessment will be more ideal to verify the reported associations.

While the results of this study, as in other studies in COPD [19], suggesting that exacerbation of underlying chronic respiratory disease could be linked to adverse renal outcomes, the presence of other systemic factors such as co‐existing HT and DM, the emerging hospital admission, and the presence and the severity of infections and the systemic administration of antibiotics that prescribed among the patients with bronchiectasis could also contribute to adverse kidney outcomes. In our study, we adjusted for the potential confounders, performed propensity score matching as well as subgroup analysis to address these issues. Nonetheless, it is worthwhile to prospectively assess this relationship by a dedicated study.

Taken together, the current study findings provide evidence of the adverse non‐respiratory outcomes related to bronchiectasis, apart from the well‐reported cardio‐respiratory outcomes, especially among patients who experienced BE. While the development of adverse kidney outcomes increases the disease burden of patients with bronchiectasis, it also confers negative clinical impacts, such as limiting the choices of pharmacotherapy for comorbidities and bronchiectasis (such as aminoglycoside), aggravating cardiovascular complications, increasing health‐care service utilization and mortality. As such, timely initiation of appropriate measures among patients who had BE should not be over‐emphasized as the benefits of these measures may extend beyond optimizing the underlying respiratory conditions but also on systemic morbidities, namely, cardiovascular and renal outcomes.

## 5 Conclusions

Renal progression and AKI were common among patients with bronchiectasis. BE was identified as the independent risk factor for adverse renal outcomes.

## Author Contributions

W.C. Kwok and D.Y.H. Yap: conception and design of study, analysis of data, writing of manuscript. T.C.C. Tam, C.K. Tsui, L. Sze Him Isaac, C.K.E. Wong, and J.C.M. Ho: revision of manuscript.

## Ethics Statement

The study was approved by the Institutional Review Board (IRB) of the University of Hong Kong and Hospital Authority Hong Kong West Cluster (UW 22‐763). Patient informed consent was waived by IRB in this retrospective study as it was a retrospective study without active patient recruitment and electronic medical record was already anonymized.

## Conflicts of Interest

The authors declare no conflicts of interest.

## Supporting information


**Figure S1** Renal progression‐free survival in a 1:1 propensity scores matched cohort of “Exacerbators” and “Non‐exacerbators” of bronchiectasis.

**Table S1** Risk factors for renal progression in patients with bronchiectasis in a 1:1 propensity score matched cohort of “Exacerbators” and “Non‐exacerbators” of bronchiectasis.
**Table S2.** Risk factors for acute kidney injury in patients with bronchiectasis in a 1:1 propensity score matched cohort of “Exacerbators” and “Non‐exacerbators” of bronchiectasis.

## Data Availability

All available data are presented in the manuscript, and no additional data will be provided. Data are not available to be shared.
