# Supplementary figures and images for: Bronchiectasis Exacerbation Increases the Risk of Adverse Renal Outcomes—Results From a Large Territory‐Wide Cohort Study
Source: Clin Respir J. 2025 Jan 11;19(1):e70029. doi: 10.1111/crj.70029 (PMC11724331; doi:10.1111/crj.70029)

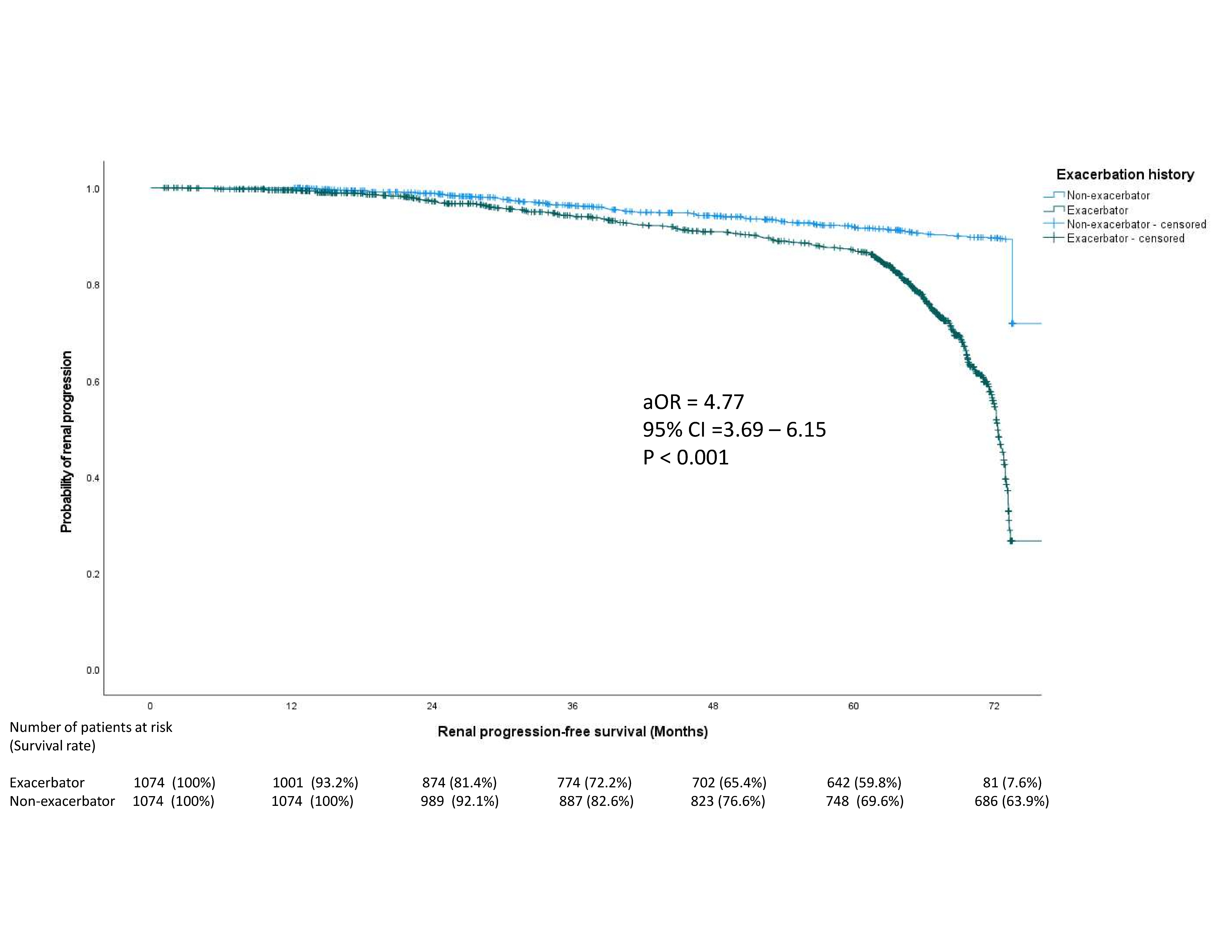

Supplement: Supplementary file 1 — Figure S1 Renal progression‐free survival in a 1:1 propensity scores matched cohort of “Exacerbators” and “Non‐exacerbators” of bronchiectasis. [file CRJ-19-e70029-s001.jpg]
