# Supplementary material for: Bronchiectasis Exacerbation Increases the Risk of Adverse Renal Outcomes—Results From a Large Territory‐Wide Cohort Study
Source: Clin Respir J. 2025 Jan 11;19(1):e70029. doi: 10.1111/crj.70029 (PMC11724331; doi:10.1111/crj.70029)
Supplement: Supplementary file 2 — Table S1 Risk factors for renal progression in patients with bronchiectasis in a 1:1 propensity score matched cohort of “Exacerbators” and “Non‐exacerbators” of bronchiectasis. Table S2. Risk factors for acute kidney injury in patients with bronchiectasis in a 1:1 propensity score matched cohort of “Exacerbators” and “Non‐exacerbators” of bronchiectasis. [file CRJ-19-e70029-s002.docx]

**Supplementary Table 1 Risk factors for renal progression in patients with bronchiectasis in a 1:1 propensity score matched cohort of “Exacerbators” and “Non-exacerbators” of bronchiectasis**

|  | **Univariate analysis** | | | **Multivariate analysis** | | |
| --- | --- | --- | --- | --- | --- | --- |
|  | **OR** | **95% CI** | **p-value** | **aOR** | **95% CI** | **p-value** |
| **Exacerbator** | 1.27 | 1.04 – 1.55 | 0.022* | 1.24 | 1.01 – 1.53 | 0.042* |
| **CHF** | 1.74 | 1.24 – 2.45 | 0.001* | 1.22 | 0.81 – 1.16 | 0.34 |
| **Stroke** | 1.68 | 1.17 – 2.40 | 0.005* | 1.16 | 0.76 – 1.76 | 0.51 |
| **DM** | 1.45 | 1.08 – 1.95 | 0.013* | 0.95 | 0.69 – 1.42 | 0.29 |
| **Baseline CCI** | 1.10 | 1.04 - 1.16 | <0.001* | 1.20 | 1.09 - 1.31 | <0.001* |
| **Annual number of bronchiectasis exacerbation from 2012 to 2016** | 1.88 | 1.33 – 2.66 | <0.001* | 1.36 | 0.93 – 1.97 | 0.11 |
| **Lower baseline eGFR** | 1.02 | 1.02 – 1.03 | <0.001* | 1.03 | 1.02 – 1.03 | <0.001* |

* = Statistically significant, IHD = ischemic heart disease; CVA = history of stroke; HT = hypertension; CHF = heart failure; DM = diabetes mellitus; CCI = Charlson comorbidity index; eGFR = estimated glomerular filtration rates

**Supplementary Table 2** **Risk factors for acute kidney injury in patients with bronchiectasis in a 1:1 propensity score matched cohort of “Exacerbators” and “Non-exacerbators” of bronchiectasis**

|  | **Univariate analysis** | | | **Multivariate analysis** | | |
| --- | --- | --- | --- | --- | --- | --- |
|  | **OR** | **95% CI** | **p-value** | **aOR** | **95% CI** | **p-value** |
| **Exacerbator** | 1.55 | 1.22 – 1.96 | 0.001* | 1.54 | 1.19 – 1.99 | 0.001* |
| ***Pseudomonas aeruginosa* colonization** | 1.65 | 1.30 – 2.09 | <0.001* | 1.19 | 0.91 – 1.54 | 0.20 |
| **IHD** | 2.18 | 1.55 – 3.06 | <0.001* | 0.86 | 0.58 – 1.28 | 0.46 |
| **CHF** | 3.07 | 2.17 – 4.35 | <0.001* | 1.04 | 0.72 – 1.20 | 0.84 |
| **Stroke** | 2.20 | 1.51 – 3.22 | <0.001* | 1.05 | 0.68 – 1.62 | 0.83 |
| **HT** | 2.51 | 1.97 – 3.19 | <0.001* | 1.21 | 0.91 – 1.60 | 0.18 |
| **DM** | 2.64 | 1.95 – 3.58 | <0.001* | 1.10 | 0.76 – 1.59 | 0.62 |
| **Baseline CCI** | 1.45 | 1.36 - 1.55 | <0.001* | 1.21 | 1.10 - 1.34 | <0.001* |
| **Annual number of bronchiectasis exacerbation from 2012 to 2016** | 2.46 | 1.55 – 3.90 | <0.001* | 1.65 | 1.01 – 2.69 | 0.045* |

* = Statistically significant, IHD = ischemic heart disease; CVA = history of stroke; HT = hypertension; CHF = heart failure; DM = diabetes mellitus; CCI = Charlson comorbidity index; eGFR = estimated glomerular filtration rates
